# A new species of the genus *Leptobrachella* Smith, 1925 (Anura, Megophryidae) from Guizhou, China

**DOI:** 10.3897/zookeys.1008.56412

**Published:** 2020-12-31

**Authors:** Jing-Cai Lyu, Liang-Liang Dai, Ping-Fan Wei, Yan-Hong He, Zhi-Yong Yuan, Wen-Li Shi, Sheng-Lun Zhou, Si-Yu Ran, Zhong-Fan Kuang, Xuan Guo, Gang Wei, Guo Yuan

**Affiliations:** 1 Guizhou Institute of Biology, Guizhou Academy of Sciences, Guiyang, 550002, China Guizhou Institute of Biology, Guizhou Academy of Sciences Guiyang China; 2 Key Laboratory for Conserving Wildlife with Small Populations in Yunnan, Southwest Forestry University, Kunming 650224, Yunnan, China Southwest Forestry University Kunming China; 3 Forestry Bureau of Congjiang County, Congjiang, 557400, Guizhou, China Forestry Bureau of Congjiang County Guizhou China; 4 Mt. Foding National Nature Reserve Bureau, shiqian, 555100, Guizhou, China Mt. Foding National Nature Reserve Bureau Shiqian China; 5 Biodiversity Conservation Key Laboratory, Guiyang College, Guiyang, 550002, China Guiyang College Guiyang China

**Keywords:** Integrated taxonomy, morphology, tadpole, vocalization

## Abstract

Asian leaf-litter toads of the genus *Leptobrachella* represent charismatic anuran diversification with 80 species, of which 25 are from China. Recent new discoveries suggest that the diversity of this genus is underestimated. Here, we describe a new species of *Leptobrachella*, *Leptobrachella bashaensis***sp. nov.** from the Basha Nature Reserve, Congjiang County, Guizhou Province, China. The new species is distinguished from its congeners by the following suite of morphological traits: small body size (SVL 22.9–25.6 mm in six adult males and 27.1 mm in one adult female); head longer than wide; dorsal skin slightly shagreened with small tubercles; creamy-white chest and belly with irregular black spots; distinct ventrolateral glands forming a white line; finger webbing and fringes absent; toe webbing rudimentary and lateral fringes narrow; iris bicolored with bright orange in upper half and silver in lower half; dorsal surface of tadpole head dark brown with small, brown, irregular spot, air sac-shaped bulges on both sides of body. The new species differs from all known congeners by an uncorrected *p*-distance of >5.3% of the 16S rRNA gene fragment examined, and the phylogenetic analysis clusters the new species with *L. maoershanensis* and *L. laui*. At present, the new species is only known from a small range of montane evergreen secondary forests in Basha Nature Reserve approximately 900 m elevation. Its natural history and conservation status are discussed.

## Introduction

At present, the megophryid genus *Leptobrachella* Smith, 1925 comprises 80 nominal species that are widely distributed from southwestern China to northeastern India, Southeast Asia and Myanmar, through mainland Indochina to peninsular Malaysia and the island of Borneo (Chen et al. 2018; Frost 2020). With the development of DNA barcoding technology and extensive fieldwork, more and more cryptic species in this genus have been reported. From 2016 to 2020, a total of 28 species were described (Eto et al. 2016, 2018; Rowley et al. 2016, 2017a, b; Yang et al. 2016, 2018; Yuan et al. 2017; Duong et al. 2018; Hou et al. 2018; Nguyen et al. 2018; Wang et al. 2018, 2019; Chen et al. 2019, 2020; Hoang et al. 2019; Li et al. 2020; Luo et al. 2020). The discovery of these species indicates that the species diversity of the genus is underestimated, and there still may be a large number of undiscovered cryptic species.

In China, the genus *Leptobrachella* is currently known to comprise 25 species, including *L. alpinus*, *L. bourreti*, *L. eos*, *L. laui*, *L. liui*, *L. mangshanensis*, *L. maoershanensis*, *L. nyx*, *L. oshanensis*, *L. pelodytoides*, *L. purpura*, *L. sungi*, *L. tengchongensis*, *L. ventripunctatus*, *L. wuhuangmontis*, *L. yingjiangensis*, *L. yunkaiensis*, *L. shangsiensis*, *L. bijie*, *L. purpuraventra*, *L. chishuiensis*, *L. feii*, *L. flaviglandulosa*, *L. niveimontis*, and *L. suiyangensis*, occurs widely in Yunnan, Guangxi and Guizhou Province (Chen et al. 2018; Wang et al. 2018; Yang et al. 2018; Chen et al. 2019; Wang et al. 2019; Amphibian China 2020; Li et al. 2020; Luo et al. 2020). More than half of the species of this genus were described in last three years, with more potential new species suggested by previous studies (Chen et al. 2018).

From 2017 to 2019, we collected a series of specimens during the field surveys in Basha Nature Reserve, Guizhou Province, Southern China. These specimens were assigned to genus *Leptobrachella* based on a combination of the following characteristics: small body size, rounded fingertips, presence of an elevated inner palmar tubercle not continuous to the thumb, absence of vomerine teeth, and vertical bars on the anterior tip of the snout. However, distinct morphological and genetic differences were found between the specimens and all recognized species. Subsequent molecular analyses confirmed that these specimens represent an as yet unknown lineage within the *Leptobrachella*. Therefore, we describe these specimens as a new species here.

## Materials and methods

### Sampling

**Figure 1.**
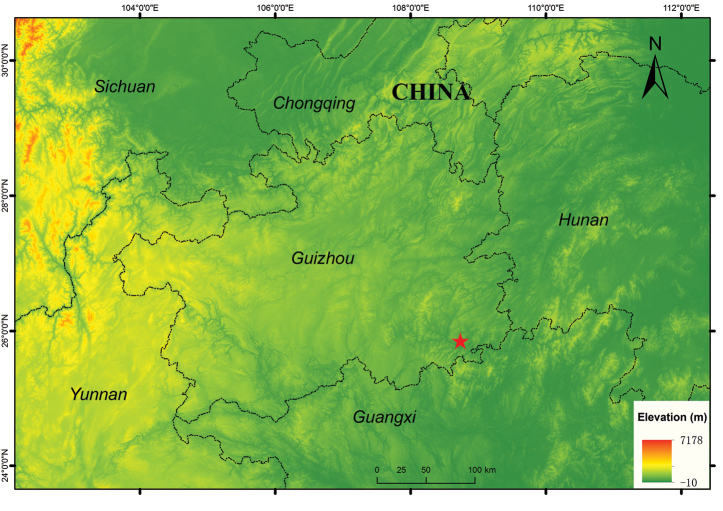
Collection locality (Basha, red star) of *Leptobrachella bashaensis* sp. nov. specimens from Basha, Guizhou, China used in this study.

A total of seven specimens were collected during fieldwork in October 2017 and March to June 2019 within Basha Nature Reserve, Guizhou Province (Fig. 1). All specimens were euthanized using chlorobutanol solution, fixed in 10% formalin for 24 hours, then stored in 75% ethanol. Liver or muscle tissues were taken from the specimens before fixing, and preserved in 95% alcohol at -20 °C. These newly collected specimens were subsequently deposited in the Museum of Biology of the Guizhou Institute of Biology, Guizhou Academy of Sciences (GIB, GAS), Guiyang, China.

### Morphology and morphometrics

All measurements were obtained to the nearest 0.1 mm (Watters et al. 2016) with digital calipers following the methods of Fei et al. (2009). The measurements of seven adult individuals were as follows:

**SVL** snout-vent length (distance from the tip of the snout to the posterior edge of the vent);

**HDL** head length (distance from the tip of the snout to the articulation of jaw);

**HDW** head width (greatest width between the left and right articulations of jaw);

**SNT** snout length (from tip of snout to anterior corner of eye);

**EYE** eye diameter (diameter of exposed portion of eyeball);

**IOD** interorbital distance (minimum distance between the inner edges of the upper eyelids);

**IND** internasal distance (minimum distance between the inner margins of the external nares);

**UEW** upper eyelid width (measured as the greatest width of the upper eyelid);

**NEL** nostril-eyelid length (distance from nostril to eyelid);

**NSL** nostril-snout length (distance from nostril to snout);

**TMP** tympanum diameter (horizontal diameter of tympanum);

**TEY** tympanum-eye distance (distance from anterior edge of tympanum to posterior corner of eye);

**TIB** tibia length (tibia length with hindlimb flexed);

**ML** manus length (distance from tip of third digit to proximal edge of inner palmar tubercle);

**LAHL** length of lower arm and hand (distance from tip of the third finger to elbow);

**HLL** hindlimb length (distance from tip of fourth toe to vent);

**FOT** foot length (from proximal edge of inner metatarsal tubercle to tip of fourth toe).

Morphometrics of a single tadpole followed Ohler et al. (2011), a total of nine morphometric characters of tadpole were measured:

**BH** maximum body height;

**BL** body length (distance from base of vent to the tip of snout);

**BW** maximum body width;

**ED** maximum eye diameter (diameter of exposed portion of eyeball);

**TH** maximum tail height (maximum height between upper and lower edges of tail);

**SS** snout to spiraculum (distance from the tip of the snout to the opening of the spiracle);

**TMW** maximum tail muscle width;

**TL** tail length (distance from base of vent to the tip of tail);

**TOL** total length (distance from the tip of snout to the tip of tail).

### DNA sequencing and analysis of sequences

Standard phenol-chloroform extraction with ethanol precipitation protocol was used to extract genomic DNA from tissues (Sambrook et al.1989). We amplified a 565~567 bp fragment 16S rRNA using polymerase chain reactions (PCRs), using the primers, and cycling conditions given by Kocher et al. (1989). Amplified PCR products were purified with enzymatic purification using exonuclease 1 and shrimp alkaline phosphatase that was incubated at 37 °C for 15 min followed by 80 °C to inactivate the enzymes. Purified PCR products were used as templates for Big Dye (Applied Biosystems, Foster City, CA) cycle sequencing. We conducted all sequencing on an ABI 3100 automated sequencers (Applied Biosystems) at Suoqin, Beijing. Considering that the morphological characters of the newly collected specimens are closest to those of *L. maoershanensis*, 19 sequences representing 16 recognized species of the seven clades belonging to the genus *Leptobrachella* from southern China (A1–A7; Chen et al. 2018) were retrieved from GenBank and included in the following phylogenetic analyses (Table 1). *Megophrys major* Boulenger, 1908 was selected as the outgroup based on the current phylogenetic hypothesis of *Leptobrachella* (Chen et al. 2018). All the sequences were aligned using MUSCLE v. 3.6 with the default settings (Edgar 2004).

**Table 1.** List of voucher specimens and GenBank accession numbers for all DNA sequences of the mitochondrial 16S rRNA gene fragments of *Leptobrachella* included in this study.

ID	Species	Locality	Voucher no.	GenBank no.
**1**	*L. aereus*	Vilabuly, Savannakhet, Laos	NCSM 76038	MH055809
**2**	*L. aereus*	Savannakhet, Laos	SAM R64242	KR018123
**3**	*L. alpinus*	Huangcaoling, Yunnan, China	KIZ046816	MH055866
**4**	*L. bijie*	Bijie City, Guizhou, China	SYS a007313	MK414532
**5**	*L. bijie*	Bijie City, Guizhou, China	SYS a007314	MK414533
**6**	*L. bourreti*	Sapa,Lao Cai, Vietnam	1999.5660	KR827860
**7**	*L. bourreti*	Bat Xat, Lao Cai, Vietnam	ZMMU-A-5636-02280	MH055872
**8**	*L. chishuiensis*	Chishui City,Guizhou Province, China	CIBCS20190518047	MT117053
**9**	*L. chishuiensis*	Chishui City,Guizhou Province, China	CIBCS20190518042	MT117054
**10**	*L. eos*	Boun Tay, Phongsaly, Laos	NCSM 80551	MH055887
**11**	*L. eos*	Boun Tay, Phongsaly, Laos	MNHN:2004.0277	JN848448
**12**	*L. feii*	Wenshan Prefecture, Yunnan Province, China	KIZ032625	MT302635
**13**	*L. feii*	Honghe, Yunnan Province, Chin	KIZ048973	MT302637
**14**	*L. firthi*	Ngoc Linh Nature Reserve, Kon Tum, Vietnam	AMS: R 176506	JQ739207
**15**	*L. flaviglandulosa*	Xiaoqiaogou Nature Reserve, Yunnan, China	KIZ016064	MT302624
**16**	*L. flaviglandulosa*	Xiaoqiaogou Nature Reserve, Yunnan, China	KIZ032626	MT302633
**17**	*L. isos*	Gia Lai, Vietnam	AMS: R 176469	KT824767
**18**	*L. khasiorum*	Khasi Hills, Meghalaya, India	SDBDU 2009.329	KY022303
**19**	*L. laui*	San zhoutian, Shenzhen, China	SYS a002540	MH055904
**20**	*L. laui*	Mt. Wutong, Shenzhen, China	SYS a003477	MH605576
**21**	*L. liui*	Mt. Wuyi, Fujian, China	SYS a002478	MH605573
**22**	*L. liui*	Mt. Huanggang, Jiangxi, China	SYS a001620	KM014549
**23**	*L. mangshanensis*	Mangshan, Hunan, China	MSZTC201702	MG132197
**24**	*L. mangshanensis*	Mangshan, Hunan, China	MSZTC201703	MG132198
**25**	*L. maoershanensis*	Maoershan, Guangxi, China	KIZ019386	KY986931
**26**	*L. maoershanensis*	Mao’er Shan, Guangxi, China	KIZ07614	MH055927
**27**	*L. maoershanensis*	Mao’er Shan, Guangxi, China	KIZ027236	MH055928
**28**	*L. bashaensis* sp. nov.	Basha Nature Reserve, Guizhou, China	GIB196403	MW136294
**29**	*L. bashaensis* sp. nov.	Basha Nature Reserve, Guizhou, China	GIB196404	MW136295
**30**	*L. minimus*	Doi Chiang Dao, Chiangmai, Thailand	THNHM07418	JN848402
**31**	*L. minimus*	Doi Suthep, Thailand	KUHE:19201	LC201981
**32**	*L. nahangensis*	Na Hang Nature Reserve, Tuyen Quang, Vietnam	ROM 7035	MH055853
**33**	*L. nahangensis*	Na Hang, Tuyen Quang, Vietnam	ZMMU-NAP-02259	MH055854
**34**	*L. namdongensis*	Thanh Hoa Provincen, Vietnam	VNUF A.2017.37	MK965389
**35**	*L. niveimongtis*	Daxueshan Nature Reserve, Yunnan, China	KIZ028276	MT302620
**36**	*L. niveimongtis*	Daxueshan Nature Reserve, Yunnan, China	KIZ028277	MT302621
**37**	*L. nyx*	Mount Tay Conn Linh, Ha Giang, Vietnam	AMNH A163810	DQ283381
**38**	*L. nyx*	Ha Giang, Ha Giang, Vietnam	ROM 36692	MH055816
**39**	*L. oshanensis*	Emei Shan, Sichuan, China	KIZ025776	MH055895
**40**	*L. oshanensis*	Emei Shan, Sichuan, China	YPX37492	MH055896
**41**	*L. petrops*	Cham Chu Nature Reserve, Tuyen Quang, Vietnam	VNMN:2016 A.06	KY459998
**42**	*L. pluvialis*	Fansipan, Lao Cai, Vietnam	ROM 30685	MH055843
**43**	*L. puhoatensis*	Pu Hu Nature Reserve, Thanh Hoa, Vietnam	VNMN:2016 A.23	KY849587
**44**	*L. puhoatensis*	Pu Hu Nature Reserve, Thanh Hoa, Vietnam	AMS:R184852	KY849588
**45**	*L. purpuraventra*	Bijie City, Guizhou, China	SYS a007081	MK414517
**46**	*L. purpuraventra*	Bijie City, Guizhou, China	SYS a007277	MK414518
**47**	*L. shangsiensis*	Guangxi Prov.,China	NHMG1401032	MK095460
**48**	*L. shangsiensis*	Guangxi Prov.,China	NHMG1401033	MK095461
**49**	*L. suiyangensis*	Suiyang County,Guizhou, China	GZNU20180606002	MK829648
**50**	*L. suiyangensis*	Suiyang County,Guizhou, China	GZNU20180606006	MK829649
**51**	*L. sungi*	Tam Dao, Vinh Phuc, Vietnam	ROM 20236	MH055858
**52**	*L. tengchongensis*	Gaoligong Shan, Yunnan, China	SYS a004598	KU589209
**53**	*L. tengchongensis*	Gaoligong Shan, Yunnan, China	SYS a003766	MH055897
**54**	*L. ventripunctatus*	Zhushihe, Yunnan, China	SYS a004536	MH055831
**55**	*L. yunkaiensis*	Dawuling Forest Station, Maoming City, Guangdong, China	SYS a004663	MH605584
**56**	*L. yunkaiensis*	Dawuling Forest Station, Maoming City, Guangdong, China	SYS a004664	MH605585
**57**	*L. zhangyapingi*	Chiang Mai, Thailand	KIZ07258	MH055864
**58**	*Megophrys major*	Kon Tum, Vietnam	AMS R173870	KY476333

Phylogenetic relationships were inferred using both maximum likelihood (ML) and Bayesian inference (BI). ML analysis was performed with RAx ML Black Box (Stamatakis et al. 2008) under the GTR + G model (1000 replicates). BI was performed using MrBayes v. 3.2.1 (Ronquist et al. 2012). The best-fit model was determined using the Akaike Information Criterion (AIC) computed with jModel Test 2 (Darriba et al.2012). Two independent runs were conducted for 30 million generations, sampling every 1000, with four independent chains and a burn-in of 25%. Convergence was assessed by checking stationary distribution and effective sample sizes (>200) using Tracer v. 1.6. (Rambaut et al.2014). We used MEGA v. 5.2 (Tamura et al. 2011) to calculate the interspecific mean uncorrected pairwise distances between the samples from Basha and its most closely related species based on the phylogenetic results. Besides, intraspeciﬁc distance was calculated among the samples from Basha.

## Results

A total of 565~567 base pairs (bp) of the 16S gene were obtained in the final alignment. The two sequences belonging to the newly acquired specimens (GenBank accession numbers MW136294 and MW136295) shared a same haplotype. The alignment contained 242 variable sites and 186 parsimony informative sites. The observed interspecific uncorrected *p*-distances between the new population from Basha and all species from clade A based on the study of Chen et al. (2018) varied from 5.3% (*L. maoershanensis*) to 15.7% (*L. firthi*) (Table 2).

**Table 2.** Pairwise genetic divergence between *Leptobrachella* species of monophyletic clades A based on uncorrected *p*-distance at a 16S rRNA fragment.

	Species	1	2	3	4	5	6	7	8	9	10	11	12	13	14	15	16	17	18	19	20	21	22	23	24	25	26	27	28	29	30	31	32	33
**1**	*L. bashaensis* sp. nov.	–																																
**2**	*L. maoershanensis*	0.053	–																															
**3**	*L. mangshanensis*	0.073	0.064	–																														
**4**	*L. liui*	0.089	0.070	0.026	–																													
**5**	*L. laui*	0.068	0.081	0.081	0.085	–																												
**6**	*L. yunkaiensis*	0.082	0.086	0.078	0.082	0.082	–																											
**7**	*L. flaviglandulosa*	0.061	0.074	0.066	0.061	0.077	0.062	–																										
**8**	*L. niveimontis*	0.112	0.117	0.131	0.108	0.116	0.113	0.087	–																									
**9**	*L. chishuiensis*	0.103	0.121	0.130	0.112	0.120	0.121	0.087	0.049	–																								
**10**	*L. namdongensis*	0.107	0.119	0.116	0.112	0.099	0.130	0.104	0.131	0.116	–																							
**11**	*L. suiyangensis*	0.120	0.130	0.148	0.139	0.138	0.130	0.112	0.057	0.053	0.143	–																						
**12**	*L. bijie*	0.116	0.134	0.135	0.117	0.121	0.134	0.095	0.053	0.015	0.125	0.057	–																					
**13**	*L. purpuraventra*	0.103	0.104	0.130	0.113	0.129	0.121	0.095	0.054	0.034	0.117	0.049	0.045	–																				
**14**	*L. shangsiensis*	0.107	0.107	0.126	0.122	0.129	0.125	0.095	0.140	0.148	0.131	0.166	0.157	0.139	–																			
**15**	*L. feii*	0.086	0.090	0.130	0.124	0.120	0.135	0.091	0.131	0.131	0.130	0.135	0.140	0.135	0.091	–																		
**16**	*L. tengchongensis*	0.098	0.103	0.108	0.095	0.086	0.125	0.099	0.103	0.111	0.070	0.119	0.119	0.102	0.116	0.121	–																	
**17**	*L. oshanensis*	0.105	0.104	0.113	0.096	0.127	0.126	0.091	0.062	0.077	0.132	0.086	0.082	0.057	0.131	0.133	0.108	–																
**18**	*L. eos*	0.107	0.107	0.125	0.108	0.115	0.107	0.082	0.042	0.069	0.121	0.073	0.073	0.053	0.134	0.139	0.094	0.061	–															
**19**	*L. bourreti*	0.060	0.067	0.080	0.062	0.075	0.073	0.047	0.025	0.032	0.081	0.040	0.043	0.022	0.077	0.069	0.063	0.043	0.025	–														
**20**	*L. alpinus*	0.095	0.117	0.122	0.116	0.107	0.113	0.074	0.053	0.042	0.126	0.049	0.057	0.049	0.140	0.109	0.107	0.074	0.057	0.030	–													
**21**	*L. zhangypingi*	0.115	0.132	0.121	0.125	0.124	0.127	0.086	0.148	0.138	0.130	0.150	0.147	0.141	0.133	0.125	0.111	0.139	0.128	0.090	0.120	–												
**22**	*L. sungi*	0.111	0.116	0.134	0.121	0.133	0.121	0.112	0.140	0.138	0.104	0.139	0.143	0.130	0.116	0.112	0.091	0.139	0.134	0.075	0.139	0.081	–											
**23**	*L. nahangensis*	0.098	0.103	0.121	0.099	0.115	0.116	0.082	0.117	0.108	0.096	0.126	0.121	0.108	0.073	0.086	0.104	0.113	0.108	0.043	0.104	0.120	0.099	–										
**24**	*L. pluvialis*	0.087	0.078	0.105	0.095	0.115	0.098	0.074	0.130	0.130	0.131	0.130	0.148	0.122	0.073	0.082	0.126	0.122	0.130	0.069	0.117	0.112	0.099	0.069	–									
**25**	*L. ventripunctatus*	0.112	0.108	0.127	0.104	0.124	0.128	0.091	0.144	0.148	0.118	0.176	0.158	0.149	0.082	0.082	0.113	0.141	0.144	0.077	0.144	0.112	0.103	0.061	0.066	–								
**26**	*L. nyx*	0.090	0.095	0.109	0.091	0.107	0.121	0.078	0.099	0.112	0.109	0.139	0.121	0.121	0.078	0.065	0.104	0.122	0.116	0.045	0.108	0.120	0.095	0.053	0.074	0.062	–							
**27**	*L. aereus*	0.086	0.086	0.117	0.099	0.119	0.134	0.082	0.126	0.113	0.113	0.139	0.122	0.122	0.082	0.049	0.116	0.129	0.134	0.065	0.108	0.124	0.098	0.061	0.074	0.068	0.046	–						
**28**	*L. miniums*	0.079	0.079	0.105	0.101	0.113	0.100	0.075	0.119	0.118	0.096	0.127	0.136	0.109	0.063	0.075	0.105	0.110	0.105	0.050	0.110	0.103	0.079	0.055	0.059	0.063	0.062	0.071	–					
**29**	*L. puhoatensis*	0.111	0.121	0.126	0.121	0.116	0.148	0.130	0.153	0.129	0.062	0.157	0.147	0.129	0.141	0.148	0.087	0.142	0.152	0.092	0.143	0.153	0.114	0.105	0.131	0.136	0.127	0.144	0.111	–				
**30**	*L. petrops*	0.129	0.124	0.117	0.109	0.125	0.140	0.118	0.150	0.143	0.086	0.143	0.153	0.134	0.154	0.139	0.082	0.149	0.134	0.094	0.139	0.152	0.126	0.118	0.134	0.150	0.123	0.140	0.115	0.082	–			
**31**	*L. khasiorum*	0.156	0.139	0.148	0.126	0.143	0.138	0.144	0.167	0.166	0.135	0.186	0.185	0.176	0.182	0.191	0.125	0.167	0.152	0.124	0.176	0.175	0.157	0.136	0.154	0.168	0.159	0.176	0.114	0.135	0.122	–		
**32**	*L. isos*	0.134	0.139	0.154	0.134	0.155	0.153	0.121	0.144	0.167	0.135	0.158	0.177	0.158	0.149	0.148	0.131	0.141	0.149	0.098	0.149	0.153	0.149	0.112	0.153	0.129	0.134	0.145	0.132	0.151	0.170	0.193	–	
**33**	*L. firthi*	0.157	0.172	0.153	0.130	0.143	0.169	0.130	0.143	0.152	0.141	0.162	0.166	0.162	0.153	0.153	0.131	0.149	0.162	0.097	0.152	0.153	0.140	0.107	0.144	0.117	0.117	0.137	0.133	0.146	0.156	0.156	0.136	–

**Figure 2.**
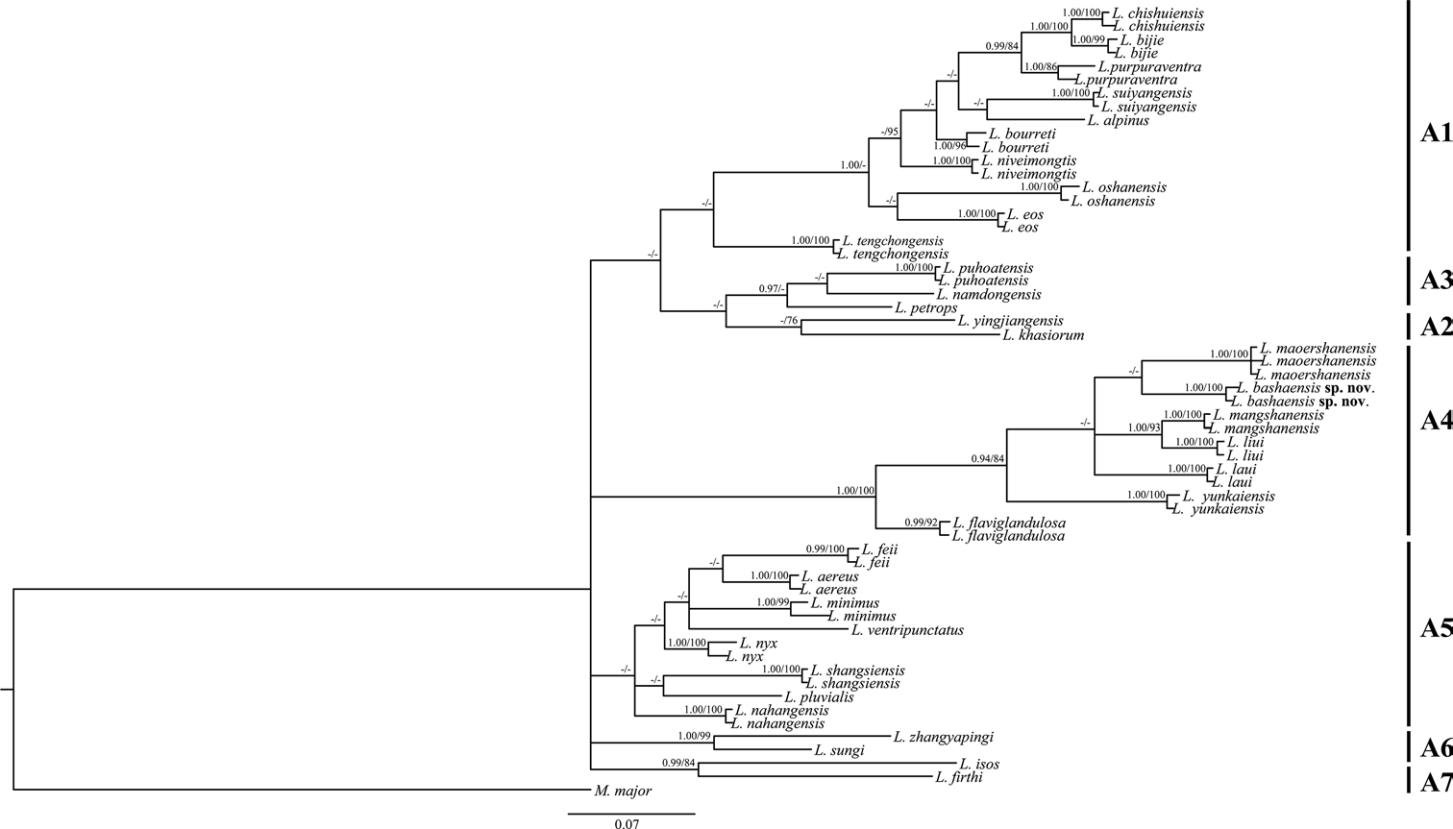
Bayesian Inference of *Leptobrachella* inferred from a 567 bp fragment of the 16S rRNA gene. Node support is indicated on branches as Bayesian posterior probabilities (BPP) (displayed values > 0.95) and Maximum Likelihood support (displayed values > 70); “-” represents low support values. The scale bar represents 0.07 nucleotide substitutions per site.

Both ML and BI (Fig. 2) analyses yielded near-identical results except for the poorly supported nodes (BS < 75% or BPP < 0.95). The relationships of the most clades (A1–A7) of *Leptobrachella* were not resolved, which is similar to previous studies (Yang et al. 2016; Yuan et al. 2017) due to limited information of 16S. The samples from Basha, Guizhou, formed a monophyletic group (Clade A4, Fig. 2) with *L. liui*, *L. laui*, *L. mangshanensis*, *L. maoershanensis*, and *L. yunkaiensis* with high support in both analyses, but the relationships among these species were not resolved. The samples from Basha formed a lineage that clustered with *L. maoershanensis*, but with low support (BPP < 95% and BS < 70%). In combination with morphological differences (see below), we conclude that the newly collected Basha specimens represent a distinct species, and we describe it herein.

### Taxonomic account

#### Leptobrachella bashaensis sp. nov.

Taxon classificationAnimaliaAnuraMegophryidae

1A5B45F4-AC3B-50D4-8942-6873D63DE751

http://zoobank.org/6257CDCB-B124-4649-BE10-9A4874C67F20

**Figure 3.**
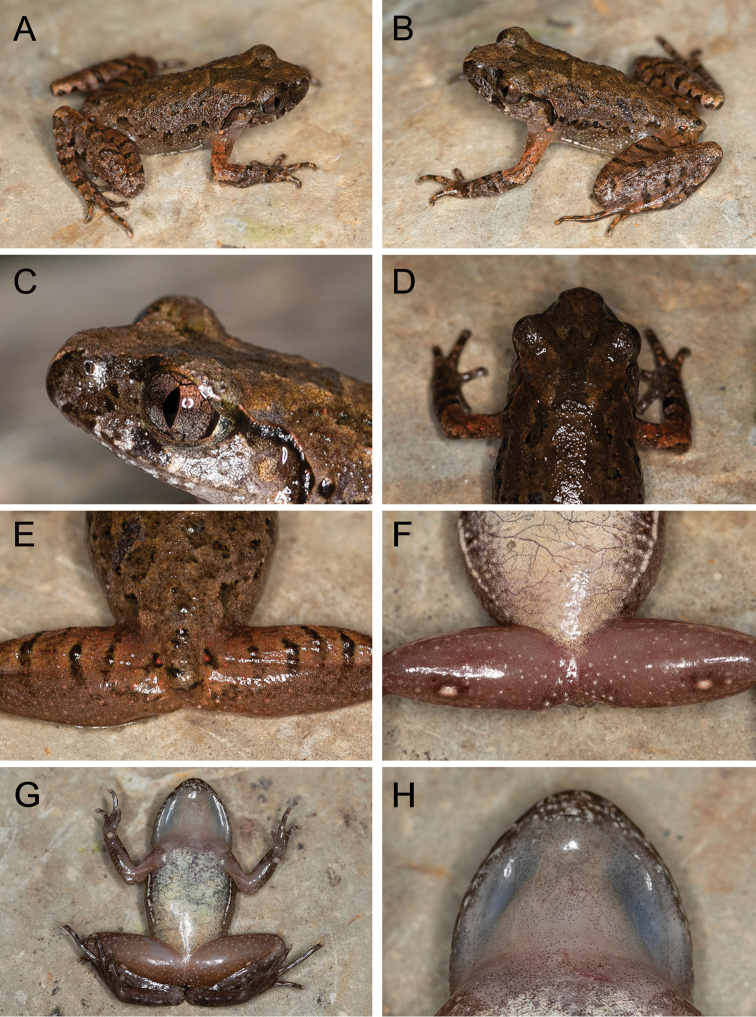
Holotype of *Leptobrachella bashaensis* sp. nov. (GIB196403) in life **A** frontolateral view **B** lateral view **C** iris coloration **D** W-shaped marking **E** dorsal view of thighs **F** posterioventral view of thighs **G** ventral view **H** throat view.

**Figure 4.**
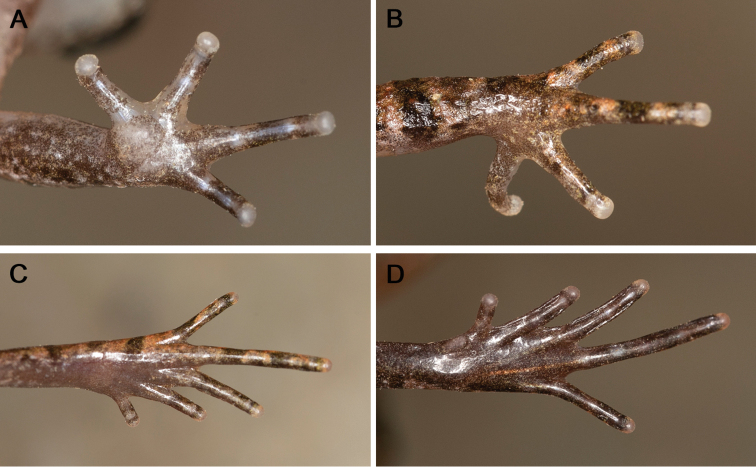
Holotype of *Leptobrachella bashaensis* sp. nov. (GIB196403) in life **A, B** ventral and dorsal view of the hand **C, D** dorsal and ventral views of the foot.

**Figure 5.**
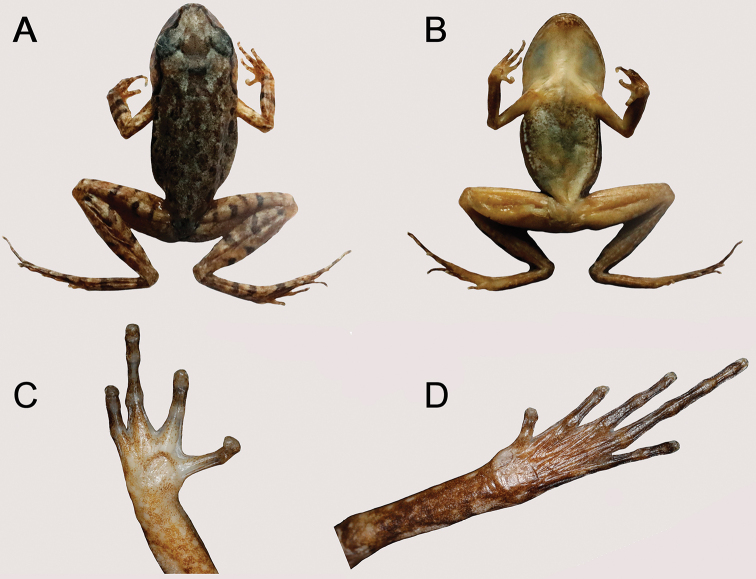
Holotype of *Leptobrachella bashaensis* sp. nov. (GIB196403) in preservation **A** dorsal view **B** ventral view **C** volar view of hand **D** plantar view of foot.

**Figure 6.**
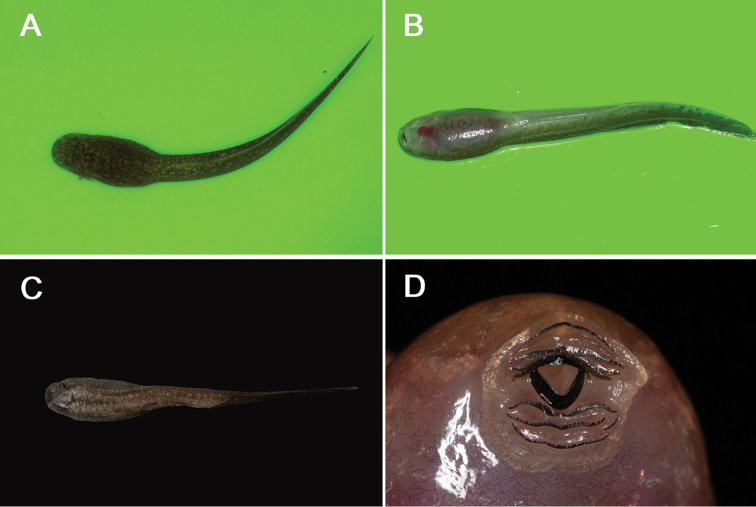
Tadpole of *Leptobrachella bashaensis* sp. nov. in preservative **A** dorsal view **B** ventral view **C** dorsal view **D** labial tooth.

**Figure 7.**
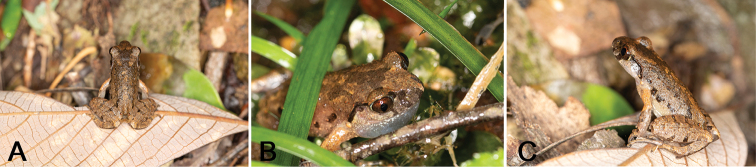
Paratypes of *Leptobrachella bashaensis* sp. nov. in life (GIB196404).

Figs 3 , 4 , 5 , 6 , 7 ; Tables 1 , 2 , 3

##### Type material.

***Holotype*.**GIB196403, adult male, from Basha Nature Reserve, Congjiang, Guizhou Province, China (25.8534°N, 108.7356°E, elevation 900 m. a.s.l.; Fig. 1), collected by Jingcai Lyu and Liangliang Dai on 4 June 2019. ***Paratypes*.**GIB196401–02, GIB196404, GIB196406–07 (five adult males) collected from the holotype locality by Jingcai Lyu on 4 June 2019.

##### Diagnosis.

The new species is assigned to the genus *Leptobrachella* on the basis of the following characters: small size, rounded fingertips, presence of an elevated inner palmar tubercle not continuous to the thumb, presence of supra-axillary, femoral and ventrolateral glands, vomerine teeth absent, tubercles on eyelids, and pale vertical bar present on anterior tip of snout (Dubois 1980, 1983; Ohler et al. 2011; Rowley and Cao 2009; Rowley et al. 2013). *Leptobrachella bashaensis* sp. nov. is distinguished from its congeners by a combination of the following morphological characters: (1) small size (SVL 22.9–25.6 mm in six adult males and 27.1 mm in one adult female), (2) head longer than wide, (3) externally distinct tympanum, (4) dorsal skin slightly shagreened with small tubercles and irregular brown stripes, (5) distinct dark spots on the flank, (6) creamy-white chest and off-white belly with irregular black spots, (7) grey-pinkish to dark brownish-violet ventral skin of limbs with numerous whitish speckles, (8) distinct ventrolateral glands, forming a distinct white line, (9) finger webbing and fringes absent, (10) toe webbing rudimentary and lateral fringes narrow, (11) longitudinal ridges under toes and not interrupted at the articulations, (12) a distinctly bicolored iris, typically bright orange in upper half, fading to silver in lower half.

##### Description of the holotype.

Adult male. SVL 24.0 mm; head slightly longer than wide (HDL/HDW = 1.10), rectangular in dorsal view (Fig. 3A, B); snout rounded in both ventral view and lateral view, protruding slightly beyond lower jaw (Fig. 3D); nostril oval, located closer to tip of snout than to eye; loreal region oblique; canthus rostralis distinct; eye large (EYE/HDL = 0.35), diameter slightly smaller than snout length (EYE/SNT = 0.88), notably protuberant in both dorsal and lateral view; pupil vertical; tympanum distinct, rounded, diameter smaller than that of eye (TMP/EYE = 0.60) (Fig. 3C); vomerine teeth absent; vocal sac openings slit-like, located posterolateral on floor of mouth; tongue long and moderately wide, with a shallow notch at posterior tip; supratympanic ridge distinct, running from eye towards axillary with raised tubercles. Forelimb long and slender, fingers long and slender, without webbing and lateral fringes; relative length of fingers II<I<IV<III; tips of fingers rounded and slightly swollen; nuptial pad absent; subarticular tubercles absent in fingers; inner metacarpal tubercle large and rounded, separated from laterally compressed and much smaller outer metacarpal tubercle (Fig. 4A, B). Hindlimb moderately long; tibia half of snout-vent length (TIB/SVL = 0.50); tibiotarsal articulation of adpressed limb reaching snout, well beyond anterior margin of eye, but not beyond snout tip; relative toe lengths I<II<V<III<IV; toe tips rounded and thickened; greatly reduced basal webbing present between all five toes; narrow lateral fringes present only on II and III toes (Fig. 4C, D); subarticular tubercles hardly discernible under toes II and III; dorsal skin slightly rough with small tubercles and irregular pustules; ventral skin smooth; oval supra-axillary gland present at forelimb base on ventral surface of axillary region (Fig. 3F); oval femoral glands distinct on posteroventral surface of thigh, closer to knee than to vent; ventrolateral glands forming a distinct line on flanks.

##### Coloration of holotype in life.

Dorsal surface of head and trunk brown with small, dark brown, irregularly shaped markings; large reverse-triangle dark brown marking between eyes, connected to the W-shaped mark between axillae (Fig. 3); supratympanic ridge present; elbow to upper arm reddish in color; fine, distinct reddish tubercles scattered on upper eyelids, dorsal surfaces of head, body, and limbs. Ventral surface of throat and belly off-white, with faint spots; chest creamy white, with irregular black spots (Fig. 3G, H); ventral surfaces of lip and limbs covered with irregular white speckles; ventral surface of thighs grey-brown with white spots. Supra-axillary gland, ventrolateral glands, and femoral glands white. Iris bicolored: bright orange in upper half, silver in lower half, with black reticulations throughout. Fine, faint transverse dark brown bars on dorsal surface of fingers and toes, lower arms, tarsus, thighs, and tibia (Fig. 4).

##### Coloration of holotype in preservation.

In preservative (75% ethanol), dorsal surface dark grey-brown, and ventral and lateral white glands not evident (Fig. 5); ventral surface of throat, chest, belly, and interior portions of arms fade to creamy white, ventral surface of thighs dark brown with white spots; bars and blotches on dorsum and limbs are dark brown and less apparent. The color of the tympanum fades to brown.

##### Tadpoles.

Brown, narrow; BH 4.4 mm; BL 14.1 mm; BW 6.2; ED 2.0 mm; TH 3.8; SS 4.9 mm; TMW 3.4 mm; TL 29.3 mm; TOL 43.8 mm. TAL/TOL = 0.67, with a I:3+3/1+1:II labial tooth row formula (Fig. 6). In life, dorsal surface of head dark brown with small, brown, irregularly shaped spot; air sac-shaped bulges on both sides of the body; upper lip and lower lip nearly round shape. Tadpoles were collected in the field from a stream surveyed on 16 October 2017 in Basha Nature Reserve by Lyu.

##### Etymology.

The specific epithet, “*bashaensis*”/Basha Zhang Tu Chan (岜沙掌突蟾 in Chinese), refers to the location where the specimens were collected, Basha Nature Reserve of Guizhou Province, China.

##### Morphological variation.

Variation in size and body proportions of the type series are shown in Table 3. Representative photographs of paratypes in life are shown in Fig. 7. In life, the dorsal surface of the head and trunk varies from olive-brown to reddish; the ventral surface of the lower jaw among these individuals varies from cream-yellow, pink, to grey; the ventral surface of chest and belly is grey-pink to white. There is variation among individuals in the shape of tubercles, pustules, black ventrolateral blotches, and dark stripes, bars on the dorsum or dorsal surface of limbs. The W-shaped marking between axillae is distinct in all individuals.

**Table 3.** Morphometric measurements (in mm) of *Leptobrachella bashaensis* sp. nov. Abbreviations are defined in the text.

Voucher number	GIB196401	GIB196402	GIB196403	GIB196404	GIB196406	GIB196407	GIB196405
Sex	Male	Male	Male	Male	Male	Male	Female
SVL	22.9	23.0	24.0	24.6	25.6	24.2	27.1
HDL	8.4	8.3	9.4	8.7	9.4	9.1	9.6
HDW	8.0	7.5	8.7	8.1	8.2	8.0	8.2
SNT	3.3	3.4	3.5	3.5	4.0	3.8	3.8
IND	2.5	2.4	2.4	2.7	2.7	2.5	2.9
IOD	3.2	3.5	3.3	3.3	3.3	3.2	3.3
UEW	2.3	2.1	2.2	2.2	2.3	2.5	2.3
EYE	2.8	2.7	2.9	3.1	3.5	3.3	3.6
NEL	1.5	1.6	1.8	2.0	1.9	1.8	2.0
NSL	1.9	1.8	1.7	1.7	1.9	1.6	1.9
TEY	0.7	0.9	1.1	0.9	1.0	1.0	1.1
ML	6.2	5.6	6.1	6.0	6.0	6.3	6.3
TMP	2.1	1.6	2.2	1.9	1.9	1.7	1.9
LAHL	11.7	10.4	10.8	10.9	11.5	11.6	11.9
HLL	37.4	37.6	40.7	40.3	38.8	39.7	41.3
TIB	12.1	11.4	12.6	12.3	12.1	13.2	12.9
FOT	11.0	10.9	11.2	11.3	10.6	11.6	11.8

##### Natural history.

**Figure 8.**
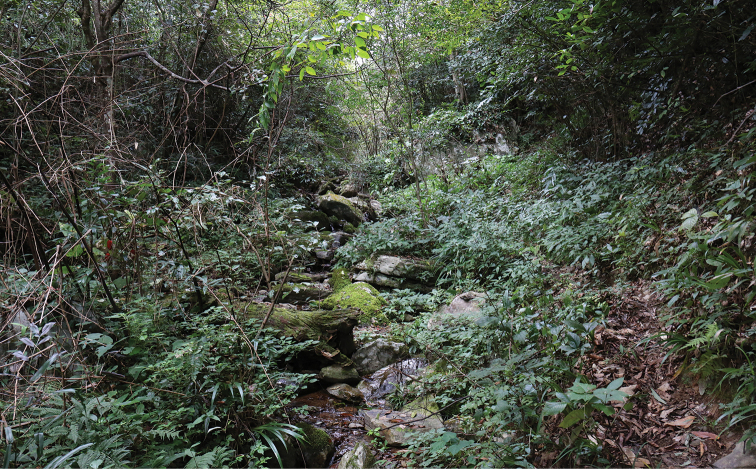
Habitat at the type locality of *Leptobrachella bashaensis* sp. nov., Basha Nature Reserve, Congjiang, Guizhou province, China.

All specimens were collected at night in small streams in Basha Nature Reserve approximately 900 m elevation (Fig. 8). Calling males were found along the streams, perching on large rocks, in rocky crevices, or under dead wood. Insect-like calls could be heard in June. The breeding season of this species is likely to occur from June to July, as females collected during these months were gravid, and males were heard calling only from June to the beginning of July. During both surveys, the number of males observed was much greater than females (males:females = 12:1).

##### Comparisons.

*Leptobrachella bashaensis* sp. nov. differs from all known *Leptobrachella* species distributed north of the Isthmus of Kra by a combination of male body size, externally distinct tympanum, and black spots on the flank, plus ventral coloration, degree of webbing and fringing on the toes, iris coloration, and dorsal skin texture.

Care was taken to differentiate *Leptobrachella bashaensis* sp. nov. from the five most morphologically and molecularly similar species, *L. maoershanensis*, *L. laui*, *L. mangshanensis*, *L. yunkaiensis*, and *L. liui* from China.

*Leptobrachella bashaensis* sp. nov. differs from its sister taxon *L. maoershanensis* by the following: smaller body size (SVL 22.9–25.6 mm in males, 27.1 mm in female vs 25.2–30.4 mm in males, 28.6 mm in female); bicolored iris with bright orange in upper half, fading to silver in lower half (vs typically bright orange-red in upper half, fading to silver in lower half); vertical and distinct narrow pupil (vs vertical and wide pupil); narrow lateral fringes present only on II and III toes (vs narrow lateral fringes present on all toes).

*Leptobrachella bashaensis* sp. nov. differs from *L. laui* in having slightly a smaller body size (SVL 22.9–25.6 mm in males, 27.1 mm in female vs 24.8–26.7 mm in males, 28.1 mm in female); bicolored iris with bright orange in upper half, fading to silver in lower half (vs uniformly coppery orange with fine black reticulations throughout); vertical and distinct narrow pupil (vs vertical and wide pupil); narrow lateral fringes present only on II and III toes (vs wide lateral fringes present on all toes); distinct black spots on the flank (vs indistinct black spots on the flank).

*Leptobrachella bashaensis* sp. nov. differs from *L. mangshanensis* by having slightly smaller body size (SVL 22.9–25.6 mm in males, 27.1 mm in female vs 22.2–27.8 mm in males, 30.2 mm in female); bicolored iris with bright orange in upper half, fading to silver in lower half (vs bright orange in upper half, greyish cream in lower half); narrow lateral fringes present only on II and III toes (vs weak lateral fringes on toes).

*Leptobrachella bashaensis* sp. nov. differs from *L. yunkaiensis* by having smaller body size (SVL 22.9–25.6 mm in males, 27.1 mm in female vs 25.9–29.3 mm in males, 34.0–35.3 mm in females); bicolored iris with bright orange in upper half, fading to silver in lower half (vs coppery orange in upper half and silver in lower half); narrow lateral fringes present only on II and III toes (vs wide lateral fringes present on all toes); distinct black spots on the flank (vs indistinct black spots on the flank).

*Leptobrachella bashaensis* sp. nov. differs from *L. liui* in having slightly smaller body size (SVL 22.9–25.6 mm in males, 27.1 mm in female vs 23.0–28.7 mm in males, 23.1–28.1 mm in females); vertical and distinctly narrow pupil (vs vertical and wide pupil); narrow lateral fringes present only on II and III toes (vs wide lateral fringes present on all toes).

## Discussion

Chen et al. (2018) suggested that *Leptobrachella* may contain hidden species diversity based on multiple nuclear DNA markers assessed using larger sample sizes, combined with morphological, nuclear gene, and bioacoustic data. With the development of DNA barcoding technology and extensive field work, more and more cryptic species in this genus have been reported. The number of *Leptobrachella* species recorded in China is now up to 25, including the new species. The discovery of this species reaffirms that the diversity within the genus is underestimated, with many species yet to be discovered.

Guizhou Province is located in the typical Karst landform area of southern China with an abundance of species diversity. So far, eight species of the genus *Leptobrachella* (*L. liui*, *L. oshanensis*, *L. pelodytoides*, *L. ventripunctatus*, *L. bijie*, *L. purpuraventra*, *L. chishuiensis*, and *L. suiyangensis*) were recorded in Guizhou province, while *L. bashaensis* sp. nov. has not been included in previous studies, which indicates that there may potentially be more new species to be found by further field research (Wu et al. 1987; Wang et al. 2019).

We observed a restricted distribution of the species in Basha Natural Reserve. We believe that *L. bashaensis* sp. nov. has a specific niche associated with small streams. The following threats were observed: reduced forest coverage, soil erosion, habitat destruction, and local people using the tadpole of *L. bashaensis* sp. nov. for food. Hence, greater protection is required for this endemic species inhabiting this high-diversity region.

## Supplementary Material

XML Treatment for Leptobrachella bashaensis
